# Identification and Biocontrol Potential of Entomopathogenic Nematodes and Their Endosymbiotic Bacteria in Apple Orchards against the Codling Moth, *Cydia pomonella* (L.) (Lepidoptera: Tortricidae)

**DOI:** 10.3390/insects13121085

**Published:** 2022-11-24

**Authors:** Asım Gümüşsoy, Ebubekir Yüksel, Göksel Özer, Mustafa İmren, Ramazan Canhilal, Mohammed Amer, Abdelfattah A. Dababat

**Affiliations:** 1 Department of Plant Protection, Faculty of Agriculture, Erciyes University, Melikgazi, 38030 Kayseri, Türkiye; 2 Department of Plant Protection, Faculty of Agriculture, Abant Izzet Baysal University, 14030 Bolu, Türkiye; 3 Department of Mechanical Engineering, National Yang Ming Chiao Tung University, Hsinchu 30010, Taiwan; 4 International Maize and Wheat Improvement Centre (CIMMYT), Emek, 06490 Ankara, Türkiye

**Keywords:** sustainable control, biological control, apple, beneficial nematodes, entomopathogenic bacteria

## Abstract

**Simple Summary:**

The codling moth, *Cydia pomonella* (L.) (Lepidoptera: Tortricidae), is a key pest in apple production. Controlling *C. pomonella* infestations with insecticides can be challenging, as it requires excessive insecticide use during the growing season. A survey of entomopathogenic nematodes (EPNs) and their endosymbionts (ESs) in apple production orchards of Kayseri Province, one of the main apple production areas of Türkiye, was conducted to develop an alternative control strategy to chemicals. Both EPNs and ESs have been studied for their potential control of *C. pomonella* larvae. The results demonstrate that EPNs and their ESs can reduce larval infestations of *C. pomonella*.

**Abstract:**

The codling moth, *Cydia pomonella* (L.) (Lepidoptera: Tortricidae), is one of the major pests in pome fruit production worldwide. Heavy treatment of the larvae of *C. pomonella* with insecticides triggered the development of resistance to many groups of insecticides. In addition, the increasing concern about the adverse effects of synthetic insecticides on human health and the environment has led to the development of sustainable and eco-friendly control practices for *C. pomonella*. The entomopathogenic nematodes (EPNs) (*Steinernema* and *Heterorhabditis* spp.) and their endosymbionts (*Xenorhabdus* and *Photorhabdus* spp.) represent a newly emerging approach to controlling a wide range of insect pests. In the present study, field surveys were conducted in apple orchards to isolate and identify EPNs and their endosymbionts and evaluate their insecticidal efficacy on the larvae of *C. pomonella*. EPNs were isolated from 12 of 100 soil samples (12%). Seven samples were identified as *Steinernema feltiae* (Filipjev, 1934) (Rhabditida: Steinernematidae), whereas five samples were assigned to *Heterorhabditis bacteriophora* (Poinar, 1976) (Rhabditida: Heterorhabditidae). The pathogenicity of the EPN species/isolates was screened on the last instar larvae of *G. mellonella*. The two most pathogenic isolates from each EPN species were tested against fifth instar larvae of *C. pomonella* under controlled conditions. The maximum mortality (100%) was achieved by all EPN species/isolates at a concentration of 100 IJs/larva 96 h after treatment. The endosymbionts of selected *H. bacteriophora* and *S. feltiae* species were identified as *Photorhabdus luminescens* subsp. *kayaii* and *Xenorhabdus bovienii*, respectively. The mortality rates ranged between 25 and 62% when the fifth larval instar larvae of *C. pomonella* were exposed to the treatment of cell-free supernatants of symbiotic bacteria. In essence, the present survey indicated that EPNs and their symbiotic bacteria have good potential for biological control of *C. pomonella*.

## 1. Introduction

Apple (*Malus* spp.) is one of the most economically important fruit crops produced widely in temperate regions of the world. Due to climate and field conditions, Türkiye is one of the leading countries in apple cultivation, with an annual production of 4.3 million tons [1]. Pest and disease management practices heavily influence the profitability of apple cultivation in orchards [2]. Of the numerous pests that infest and damage apple orchards, the codling moth, *Cydia pomonella* (L.) (Lepidoptera: Tortricidae), is one of the major pests of apple orchards worldwide because the larvae directly feed on fruits. The adults of *C. pomonella* lay their eggs on the surface of apple fruits after emerging in late spring. The hatching larvae begin feeding by boring into fruits and forming galleries towards the seed chamber of the fruit [3]. Because the internal feeding damage of larvae renders fruits unmarketable, the control of larvae is of great importance for the management of *C. pomonella*. In the absence of appropriate control measures, apple orchards experience serious yield losses, rendering more than 50% of fruits unmarketable [4].

Among the main strategies used in Türkiye to combat codling moths are synthetic insecticides. Insecticides are normally applied more frequently than once a week to prevent neonate larvae from tunneling into fruits following the emergence of adults during the growing season. As a consequence of the intensive use of insecticides, *C. pomonella* has developed resistance to almost all classes of insecticides, leaving their residues on apple fruits that exceed maximum residue levels (MRLs) [5,6,7,8,9,10]. In addition to dependence on insecticides, these MRLs have led to several environmental and health problems [11]. Therefore, the assessment and development of biologically based alternatives to control *C. pomonella* have been one of the focuses of agricultural research in the last decades [12,13]. Of alternative control strategies for *C. pomonella*, entomopathogenic nematodes (EPNs) (*Steinernema* spp. and *Heterorhabditis* spp.) have attracted great attention due to their biocontrol potential and environmental safety [14]. Entomopathogenic nematodes can reduce the number of insecticide applications and the production costs while providing environmentally safe control. Infective juveniles (IJs) of EPNs are a non-feeding and free-living stage that is able to seek out a potential host in cryptic habitats. Once IJs encounter a host, they penetrate its body using body openings and thin cuticles [15]. The IJs initiate the infection process by releasing their mutualistic bacteria (*Xenorhabdus* and *Photorhabdus* spp.) into insect hemolymph. The symbiotic bacteria multiply rapidly and serve as a nutrition source for the IJs along with host tissues. The host generally dies from septicemia within 48 h after infection due to the toxin complexes and killer proteins produced by the endosymbionts of EPNs [16]. After a few generations, large numbers of IJs abandon the resource-depleted host cadaver to search for a new host [17].

Although EPNs are soil-originated organisms, their ability to find and infect insect pests in cryptic habitats such as crevices in wood and litter at the base of trees where the fifth instar larvae of *C. pomonella* choose to pupate makes them a perfect candidate for the biological management of orchards [18]. Earlier laboratory and field studies showed that first and fifth instar *C. pomonella* larvae were susceptible to EPNs [19,20,21,22]. However, to our knowledge, there is no study assessing the effectiveness of EPNs recovered from apple orchards on the larvae of *C. pomonella*. The virulence of EPNs varies greatly among species/isolates depending on the host-finding behaviors, adaptation and penetration capabilities, host specialization, and symbiotic bacteria [23,24]. Therefore, the isolation of native EPN species from orchards and the determination of their virulence on the target host are of crucial importance for the success of EPNs applications. *Photorhabdus* and *Xenorhabdus* spp., the endosymbionts of *Heterorhabditis* and *Steinernema* spp., are Gram-negative bacteria that play a key role in the pathogenicity of EPNs by releasing a wide range of seconder metabolites into host hemolymph, which is lethal to host insects [16]. Numerous studies have revealed that toxin complexes of *Photorhabdus* and *Xenorhabdus* spp. bacteria were highly lethal to tested insects and offered an alternative in the control of many economically important pests [25,26,27,28]. However, to date, no study has been conducted to evaluate the cell-free supernatants of *Photorhabdus* and *Xenorhabdus* spp. of bacteria, which contain insect-killer proteins, proteases, lipases, and toxic compounds, to insects [29]. Thus, the aim of this study was to examine the diversity and the distribution of EPNs in apple-growing areas in Kayseri Province and evaluate their effectiveness and their endosymbiotic bacteria on fifth larval instars of *C. pomonella* under controlled conditions.

## 2. Materials and Methods

### 2.1. Soil Sampling

Surveys were conducted in apple orchards between 2021 and 2022 from major apple-growing areas of Kayseri. Soil samples were taken randomly from crown projection areas of apple trees at each sampling site using a hand shovel following rainy days in the spring months (April, May, and June). For each orchard, 5–12 soil samples that consisted of 250 g soil subsamples were collected according to the number of trees in each orchard. All subsamples were pooled together, and 2 kg of a mixture of subsamples in plastic bags was brought to the Entomology Laboratory of Erciyes University in a cool box (12–15 °C). A total of 100 soil samples were taken (Table 1).

### 2.2. Isolation of Entomopathogenic Nematodes

Entomopathogenic nematodes were isolated from soil samples using an insect-baiting technique [30,31]. *Galleria mellonella* (L.) (Lepidoptera: Pyralidae) larvae were reared under laboratory conditions (25 ± 2 °C and 60% RH) and the last instar larvae were used as the insect bait [32]. After sieving thoroughly using 1 cm mesh, subsamples of 250 g of soil were put into a clean plastic container (Ø12 cm and 10 cm height) with 10 larvae, and containers were covered with perforated lids. Then, containers were incubated under controlled conditions for 10 days (25 ± 2 °C and 60% RH). The dead and live larvae were checked at 2-day intervals during the incubation period. The dead larvae showing typical signs of nematode infection (soft body and creamy beige or red pigmentation) were placed individually in modified White traps for the emergence of IJs [33]. After washing with sterile water several times, the emerged IJs were applied to eight *G. mellonella* larvae in Petri dishes to verify Koch’s postulates for pathogenicity, and dead larvae were re-transferred to modified White traps to obtain pure IJs populations [34]. The newly emerged IJs were rinsed with sterile water and stored horizontally at 11 °C in flasks (250 mL).

### 2.3. Identification of Nematodes

The morphological and/or morphometric identification of EPN species was carried out using infective juveniles as suggested by [35,36]. The stock cultures of IJs from each population were applied to ten last instar *G. mellonella* larvae (2000 IJs/mL) in a Petri dish (100 × 15 mm) lined with two filter papers and incubated under controlled conditions (25 ± 2 °C and 60% RH). Dead larvae were transferred to the modified White traps and twenty IJs specimens from each EPN population were used for morphometric measurements. Prior to measurements, the IJs were killed by heat (60 °C) in Ringer’s solution and placed in TAF (triethanolamine-formalin fixative) [34,37]. Then, the specimens were mounted on microscope slides in a drop of pure glycerin and covered with a coverslip. Observations were made using an MC100 spot microscope equipped with digital image software (Axioskop; Zeiss, Oberkochen, Germany) (Table 2).

Molecular characterization of EPN isolates was performed based on sequencing of the internal transcribed spacer (ITS) of ribosomal DNA. The isolation of DNA was performed on 8–10 newly emerged, live IJs from each EPN population following the method described by [38]. The ITS region was amplified by a total of 50 μL PCR reaction mixture consisting of 2 μL DNA, 22 μL ddH2O, 25 μL of Dream Taq PCR Master Mix (Thermo Fischer Scientific, Waltham, MA, USA), 0.5 μM of F194 (5′-CGT AAC AAG GTA GCT GTA G-3′), and 5368r (5′-TCC TCC GCT AAA TGA TAT G-3′) primers [39,40]. Thermo-cycling was performed in a T100 thermal cycler (Bio-Rad, Hercules, CA, USA) and programmed for initial denaturation at 94 °C for 3 min, followed by 35 cycles at 94 °C for 1 min, extension at 72 °C for 1 min, and the final extension at 72 °C for 10 min. The resulting products were purified and sequenced in both directions using the primers referred to above (Macrogen Inc., Seoul, Korea). The obtained sequences were aligned using the MegAlign module of DNASTAR software, version 7.1.0 (DNASTAR Inc., Madison, Wisconsin, USA) and compared to the sequences deposited in the GenBank. Accession numbers submitted to the GenBank database were given in Table 3.

### 2.4. Pathogenicity Screening on Galleria mellonella Larvae

The pathogenicity of obtained EPNs was assessed on last larval instar of *G. mellonella* in Petri dishes under laboratory conditions (25 ± 1 °C, 65 ± 5% RH, and 16L: 8D h photoperiod). The IJs of EPN isolates were applied at the concentration of 100 IJs/Petri dish. Based on the mortality rates 72 h after treatment, the most efficient two isolates of each EPN species were selected to further evaluate their pathogenicity against the larvae of *C. pomonella*.

### 2.5. Cydia pomonella

Different larval instars of *C. pomonella* were collected from infested apple fruits in large numbers from apple orchards in Kayseri Province during the summer of 2021. The larval instars were reared on apple fruits in cages (60 × 60 × 60 cm) under controlled conditions (25 ± 1 °C, 65 ± 5% RH, and 16L: 8D h photoperiod). The emerging adults from each population were collected using a suction trap and sent to an entomologist for identification (Prof. Halil KÜTÜK, Abant Baysal University). The 5th instar larvae feeding within fruits were gathered and grouped together. The healthy larvae were included in the bioassays.

### 2.6. Isolation and Identification of Symbiotic Bacteria

The bacterium was isolated using approximately 500 freshly emerged IJs of each isolate. After being surface-sterilized in a sterile Ringer’s solution comprising NaClO (10% *w*/*v*) for 10 min, the IJs were washed several times with sterile Ringer’s solution and crushed in 1 mL of sterile phosphate-buffered saline. Then, 10 μL of the suspension was streaked onto NBTA medium [16]. After an incubation period of 48 h at 28 °C, primary variants were re-streaked onto the NBTA plates to obtain pure bacterial colonies. Then, each purified bacterial colony was harvested and subjected to DNA extraction using the GeneMATRIX Tissue and Bacterial DNA Purification Kit (EURx) according to the manufacturer’s instructions. The 16S ribosomal RNA (rRNA) gene was amplified and sequenced according to primers suggested by [41,42]. The PCR conditions were set as an initial denaturation at 94 °C for 2 min, followed by 34 cycles of denaturation at 94 °C for 1 min, annealing at 52 °C for 30 s, and extension at 72 °C for 1 min, followed by a final extension at 72 °C for 10 min. PCR products were checked by electrophoresis to verify amplification products and sequenced by Macrogen, Inc. (South Korea). The nucleotide sequences of the isolates/strains were checked and manually corrected. The arrangement of nucleotides in ambiguous positions was corrected by comparisons of the sequences generated from both the forward and reverse primers using MEGA X software [43]. The novel sequences were compared with the sequences registered in GenBank based on nucleotide similarity and deposited in GenBank with Accessions Nos. OP642365 (*X. bovienii* A94 strain), OP630600 (*X. bovienii* A54 strain), OP630601 (*P. luminescens* subsp. *kayaii* A8 strain), and OP642366 (*P. luminescens* subsp. *kayaii* A9 strain). 

### 2.7. Preparation of Cell-Free Bacterial Supernatants

Erlenmeyer flasks (250 mL) containing 100 mL Luria–Bertani (LB) broth (Merck, Darmstadt, Germany) were inoculated with a loopful of the pure bacterial colonies of each isolate and incubated on a rotary incubator at 150 rpm for 144 h (28 °C, 20% RH in the dark) [16,44]. The broth suspensions of bacteria cultures were then poured into 50 mL Falcon tubes. The cell-free supernatants of the solutions were separated by centrifuging the bacterial suspension at 20,000 rpm for 15 min at 4 °C and filtering twice through a 0.22 μM Millipore filter (Sigma–Aldrich). The leakage of bacterial cells was checked by streaking the final cell-free supernatant solutions onto NBTA agar.

### 2.8. Virulence Tests on Cydia pomonella Larvae

Obtained EPN isolates were tested for their pathogenicity against the last instar larvae of *C. pomonella* in Petri dishes lined with two filter papers. The IJs suspended in 1 mL of tap water from each isolate were inoculated into Petri dishes at concentrations of 10, 25, 50, and 100 IJs/Petri dish, and 10 larvae were added to each Petri dish. A button-sized apple (Approximately 1 cm^2^) was provided for the larvae as food. Then, Petri dishes were incubated under controlled conditions (25 °C, 60% RH, and 16:8 h of L/D) after sealing with a parafilm. The larval mortality was recorded daily for three days, and dead larvae were dissected under a stereo microscope to check the nematode infection. Control groups were treated with tap water. Each treatment consisted of four replicates and 10 larvae were used for each replicate. The bioassays were conducted twice under the same conditions. Only one-week-old IJs were included in the pathogenicity bioassays. Based on the mortality rates obtained on the 3rd day after treatment (DAT), the symbiotic bacteria of the most pathogenic two isolates of EPN species were selected for further evaluation of their effectiveness against 5th larval instar larvae of *C. pomonella*.

### 2.9. Evaluation of Pathogenicity of Symbiotic Bacteria

The cell-free supernatant solutions were evaluated against ten 5th instar larvae of *C. pomonella* in contact treatments under controlled conditions. A 1 mL aqueous suspension of cell-free supernatants was applied directly onto each larva with the help of a mini spray bottle (50 mL) (Ø 0.5 mm nozzle) and larvae were placed into Petri dishes (Ø 9 cm) individually containing two filter papers and a button-sized apple. The Petri dishes were covered and sealed with parafilm. The Petri dishes were kept at 25 °C, 60% RH, 14 h light, and 10 h darkness for three days and larval mortality was checked daily. In control treatments, larvae were treated with nutrient broth only and the rest of the procedure was repeated. Each treatment consisted of four replicates and 10 larvae were used for each replicate. The bioassays were conducted twice under the same conditions. Only two-week-old cell-free supernatants were included in the bioassays, and they were stored at 9 °C until the bioassays were performed.

### 2.10. Statistical Analysis

No mortality occurred in control treatments and mortality data were subjected to normality tests and were arcsine transformed. All statistical analyses were carried out using IBM SPSS Statistics, version 20.0 for Windows (SPSS Inc., Chicago, IL, USA). Significant differences between treatments were determined by factorial repeated measures ANOVA using a general linear model. The mean differences were grouped using Tukey’s multiple range tests (*p* ≤ 0.05).

## 3. Results

### 3.1. Survey and Identification of EPNs

**Figure 1:**
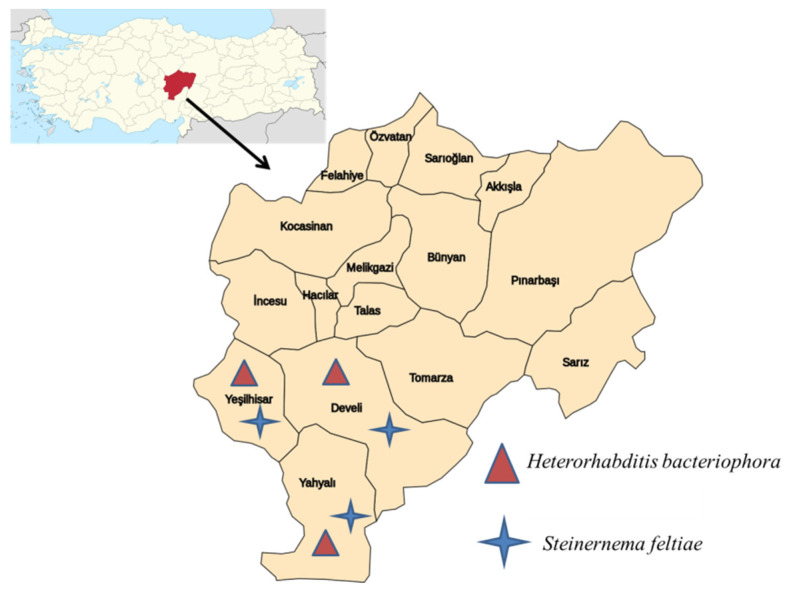
Map of Kayseri Province showing districts where entomopathogenic nematodes were found.

Out of the 100 soil samples examined for the presence of EPNs, 12 samples were positive (Table 1). Recovery rates of EPNs in the sampled districts ranged between 10.0 and 14.2%. The highest occurrence rate of EPNs (14.2%) was obtained from the Yeşilhisar district, which is the biggest apple cultivation area in Kayseri. Two EPN species were obtained from all districts. However, *Heterorhabditis bacteriophora* (Poinar, 1976) (Rhabditida: Heterorhabditidae) was more prevalent in the Develi district, whereas *Steinernema feltiae* (Filipjev, 1934) (Rhabditida: Steinernematidae) was more common in Yeşilhisar (Figure 1).

Using the taxonomic keys from [36], seven isolates were classified as *S. feltiae*, whereas five isolates belonged to *H. bacteriophora*. The morphometric measurements of IJs of *S. feltiae* and *H. bacteriophora* indicated a remarkable similarity to those described by [35,36] in terms of the most morphometric characteristics (Table 2). The morphological identification of EPNs was also confirmed by the nucleotide sequences of the ITS region of isolates. The DNA fragments of the ITS region were between 770 and 820 base pairs long. The ITS sequences of obtained isolates showed a 99% nucleotide similarity with those of reference isolates deposited in GenBank (Table 3).

### 3.2. Pathogenicity Screening on Galleria mellonella Larvae

The results showed that all isolates induced significant mortality of G. mellonella larvae (24 h: Df: 11, F-value: 43.111, and *p* = 0.000; 48 h: Df: 11, F-value: 38.291, and *p* = 0.000; 72 h: Df: 11, F-value: 6.652, and *p* = 0.000). The mortality rates ranged between 60 and 100% 72 h after treatment (Table 4). The most efficient isolates of *S. feltiae* and *H. bacteriophora* 72 h after treatment were *S. feltiae* A54 and *H. bacteriophora* A9, and these caused 100 and 96% mortality, respectively.

### 3.3. Susceptibility of Cydia pomonella Larvae to Selected Isolates

Statistical analysis revealed that the susceptibility of *C. pomonella* larvae was affected by primary factors and their associated interactions, except for the interaction of nematodes–concentrations (N*C) and nematodes–concentrations–exposure time (N*C*t) (Table 5). Mortality rates tended to increase as exposure time and IJs concentrations increased. The larvae of *C. pomonella* were susceptible to all selected EPN species/isolates and mortalities over 50% were obtained even at the lowest concentration (10 IJs/larva). Maximum mortality (100%) was achieved by only *H. bacteriophora* isolates (H. b. A8 and H. b. A9) at a concentration of 50 IJs/larva. At the lowest exposure time (24 h), *H. bacteriophora* A8 was the only isolate that caused 100% mortality at the concentration of 100 IJs/larva. However, all EPN species/isolates induced 100% mortality 96 h after treatment (Table 6).

### 3.4. Identification and Evaluation of the Symbiotic Bacteria of Selected Isolates

The bacterial associates of A94 and A54 isolates of *S. feltiae* and A8 and A9 isolates of *H. bacteriophora* were classified as X. bovienii and P. luminescens subsp. kayaii, respectively. Statistical analysis demonstrated that the cell-free supernatant of tested symbiotic bacteria species/strains and exposure time had a significant influence on the mortality rates of *C. pomonella* (Table 7). *Xenorhabdus bovienii* strains generally induced greater mortality at all exposure times compared with P. luminescens subsp. *kayaii* strains. *Xenorhabdus bovienii* strains were able to cause mortalities over 50% only 72 h after treatment. The highest mortality (62%) was achieved by the X. bovienii A94 strain (Table 8).

## 4. Discussion

In the present study, the natural occurrence and distribution of EPNs in apple orchards of Kayseri Province, Türkiye, were determined in order to evaluate the biocontrol potential of EPNs and their bacterial associations against the larvae of *C. pomonella*. Two EPN species were isolated from twelve EPN populations with a recovery rate of 12%. Although the recovery rate of EPNs varied greatly in earlier surveys of EPNs in the world (0.2% to 70%) [45,46,47], the recovery rate found (12%) in this study was quite similar to those countries in the Mediterranean region such as in Italy (13.8%) [48], in Cameroon (10%) [49], and in Egypt (9.5%) [50]. However, lower and higher prevalence of EPNs were also reported in different countries [51,52]. The difference in recovery rates of EPNs may be associated with different surveying times and methods, and monoculture crop production. Kary et al. [52] reported that sampling times had a significant effect on the recovery of EPNs and the highest recovery frequency was generally obtained in spring (April and May). *Galleria mellonella* is the most used insect in the isolation and pathogenicity testing studies of EPNs due to its susceptibility to EPNs and convenience for in vitro mass culture. However, EPNs differ in host and habitat preference, and this may be another factor behind the varying recovery rates of EPNs [46,52,53,54]. 

Although the survey area in this study was limited to apple orchards, seven *S. feltiae* and five *H. bacteriophora* populations were obtained. These results agree with most EPN surveys conducted in Türkiye with similar climatic conditions [55,56]. *Steinernema feltiae* and *H. bacteriophora* are the most frequently detected EPN species in Türkiye and other parts of the world, respectively [57,58,59,60]. However, some studies reported a higher occurrence of *H. bacteriophora* than *S. feltiae* from neighboring regions with similar climatic and geographic conditions in Türkiye [61,62]. Although both EPN species have been found in different climatic zones, *S. feltiae* was more dominant in cold and continental climate conditions, whereas *H. bacteriophora* was generally isolated in higher frequencies from tropical and subtropical areas [51,63,64]. The diversity and distribution of EPNs are also related to the presence of suitable insect hosts as well as climatic and environmental conditions. This might be another factor affecting the distribution of EPN species in varying frequencies on Earth [65].

The morphometric measurements of the IJs of *S. feltiae* and *H. bactriophora* isolates showed close similarity with the findings of Nguyen and Hunt [36] and Yüksel and Canhilal [45]. However, they also differed in several morphometric features such as body and tail length, and maximum body width reported by Stock et al. [59] and Laznik et al. [60]. The differences in these measurements might be due to habitat and intraspecific variations among the geographical isolates of EPN species as reported in earlier studies [66,67,68,69]. Therefore, in addition to morphometric measurements, the isolates were further confirmed by using molecular methods based on the nucleotide sequences of the ITS region.

Pathogenicity screening of high numbers of candidate biological control agents is a crucial step to revealing the potential of EPN species/isolates and also helps save resources and time before initiating field evaluations [69]. In the present study, preliminary tests were performed on the last instar larvae of *G. mellonella*, and variable mortalities were obtained after 72 h of exposure. Differences in larval mortality among the same EPN and symbiotic bacteria species can be explained by the secondary metabolites that are released by the symbiotic bacteria. Hasan et al. [70] tested the toxicity of different *X. nematophila* on *Spodoptera exigua* (Hübner) (Noctuidae: Lepidoptera) and *Tenebrio molitor* (L.) (Coleoptera: Tenebrionidae). They reported a variation in associated secondary metabolites’ pathogenic and immunosuppressive activities, which is in line with our study. In addition, a great variation in chemical components among the six bacterial strains of *X. nematophila* was reported by Hasan et al. [70]. Different efficacies of the same EPN and symbiotic bacteria species might be due to variations in chemical components and toxin complexes released by symbiotic bacteria. The most virulent EPN species/isolates on *G. mellonella* were further tested on the last instar larvae of *C. pomonella*. The results revealed that all selected EPN species/isolates were highly pathogenic to *C. pomonella* larvae. However, at concentrations of 50 IJs/larvae, *H. bacteriophora* isolates were more virulent than *S. feltiae* isolates and induced maximum mortality (100%) 72 h after treatment. Contrary to *Steinernema* species, *Heterorhabditis* species possess an additional dorsal tooth that facilitates penetration into a host’s body [71]. In addition, the IJs of *Heterorhabditis* species have a smaller body size than *Steinernema* species and this might have enabled them to more easily penetrate the host’s body using natural openings [72]. Lacey and Unruh [73] tested the susceptibility of *C. pomonella* larvae against *S. carpocapsae*, *S. riobrave*, and *H. bacteriophora* at 50 and 100 IJs/cm^2^ and reported that *H. bacteriophora* was more efficient than *S. riobrave*. However, *S. carpocapsae* performed better than *H. bacteriophora*. In another study conducted by Yağci et al. [22], *S. carpocapsae* and *S. feltiae* exhibited superior virulence to *H. bacteriophora* against the first larvae of *C. pomonella*, which is contradictory to the findings of this study. In a previous study evaluating the field performance of different EPN species against *C. pomonella* larvae, *S. feltiae* and *S. carpocasae* caused similar mortalities at concentrations of 50 IJs/mL [12]. The differences in the larval mortalities may be explained by the variation in the experimental setups such as IJs concentrations, application environment, and tested larval instars as well as different environmental conditions. The virulence of EPN species/isolates varies greatly depending on the host-seeking behaviors, adaptation capabilities, host specificity, and symbiotic association of IJs [23,59,65]. Among these factors, symbiotic bacteria of EPNs play a crucial role in the pathogenicity process by releasing a wide range of secondary metabolites into host hemolymph [29]. Therefore, in the present study, the cell-free supernatants of bacterial symbionts of the most pathogenic EPN species were also evaluated on *C. pomonella* larvae. To our knowledge, this is the first report on the insecticidal effect of the cell-free supernatants of *Xenorhabdus* and *Photorhabdus* bacteria species on *C. pomonella* larvae.

Based on the identity of the 16S rRNA sequence and BLAST analysis, the symbiotic bacteria of selected *S. feltiae* and *H. bacteriophora* isolates were identified as *Xenorhabdus bovienii* and *Photorhabdus luminescens* subsp. *kayaii*, respectively. The results revealed that the cell-free supernatants of *X. bovienii* and *P. luminescens* subsp. *kayaii* strains were lethal to the fifth instar larvae of *C. pomonella* in the contact efficacy bioassay. However, *X. bovienii* strains exhibited superior mortality against *C. pomonella* larvae causing mortalities over 50% 72 h after treatment. The insecticidal effect of *Xenorhabdus* and *Photorhabdus* bacteria species has been shown against different insect pests in earlier studies and varying degrees of pathogenicity have been reported. Mahar et al. [74] reported that the cell-free supernatant application of *X. nematophila* resulted in 95% mortality in the larvae of *Spodoptera exigua* (Hübner) (Noctuidae: Lepidoptera). In another study, the larvae of *Earias vittella* (F.) (Lepidoptera: Noctuidae) were exposed to the cell-free supernatants of *X. nematophila* and *P. luminescent*, and the mortality rates varied between 65 and 70% 72 h after treatment [75]. Vicente-Díez et al. [76], in their study, tested the efficacy of bacterial secretions of *X. bovienii* on the third larval instar of *Lobesia botrana* (Den. & Schiff.) (Lepidoptera: Tortricidae) and reported 50% mortality 3 days after treatment which is in line with our study. In contrast to the aforementioned studies, Shawer et al. [77] reported lower larval mortality (24%) in the larvae of *Drosophila suzukii* (Matsumura) (Diptera: Drosophilidae) after treatment with *P. luminescens* supernatant. The variation in the mortality rates can be explained by the different toxin complexes produced by *Xenorhabdus* and *Photorhabdus* species/strains as well as differences in the experimental design and larval instars of tested insects [78]. Toxin complexes and secondary metabolites play a major role in the suppression of the host immune system as well as toxicity to the host intestine [76,77,78]. Earlier studies reported that bacterial toxin complexes such as Tca, Tcb, and Tcc toxins produced by *P. luminescens* were more toxic to the tested insect when digested orally [25,26,27,78,79,80,81]. However, in the present study, cell-free supernatants were sprayed directly onto *C. pomonella* larvae, and this may have restrained the effectiveness of *P. luminescens* subsp. *kayaii* strains. Similar to our findings, the direct application of cell-free supernatants proved to be lethal against *Earias vittella* (F.) (Lepidoptera: Noctuidae) [75], *Aphis gossypii* (Glov.) (Hemiptera: Aphididae) [82], *Macrosiphum rosae* (L.) (Hemiptera: Aphididae) [83], and *Philaenus spumarius* (L.) (Hemiptera: Cercopidae) [76]. The results of the aforementioned studies indicated that secondary metabolites of different *Xenorhabdus* and *Photohabdus* species were able to penetrate through the cuticle and cause remarkable mortalities against tested insects with a varying degree of pathogenicity [44,75,76,83]. Chitinase protein secreted by *Xenorhabdus* and *Photorhabdus* bacteria plays a key role in the virulence of symbiotic bacteria by both degrading the cuticles of insects and accelerating the binding process of toxins to target sites [84,85]. The results suggest that toxic metabolites including chitinase protein are present in the cell-free supernatant of symbiotic bacteria and are responsible for mortality. In addition, Hasan et al. [70] reported that the chemical composition of cell-free supernatants produced by different bacterial strains exhibited great variation. This might be another factor causing varying mortalities in *C. pomonella* larvae by different strains of the same symbiotic bacteria.

## 5. Conclusions

Surveyed apple orchards yielded twelve EPN isolates belonging to *S. feltiae* and *H. bacteriophora* species. The two most pathogenic isolates of EPN species on *G. mellonella* larvae were further tested for their biocontrol potential on the fifth larval instar of *C. pomonella*. Selected EPN isolates achieved mortalities over 80% at 50 IJs/larva 48 h after treatment. Bacterial associates of selected EPN isolates were identified as *X. bovienii* and *P. luminscens* subsp. *kayaii*. Direct exposure to the cell-free supernatants of *X. bovienii* and *P. luminscens* subsp. *kayaii* strains were lethal to the fifth larval instar of *C. pomonella*, and the highest mortalities were obtained from *X. bovienii* strains 72 h after application. The results revealed that selected EPN species/isolates and their enteric bacteria have potential in the control of *C. pomonella*. The results also indicate that the cell-free supernatants of *X. bovienii* and *P. luminescens* subsp. *kayaii* strains can be utilized separately from their nematodes against *C. pomonella* larvae. However, further studies are required to reveal the field performance of tested EPN species/isolates and their endosymbiotic bacteria.

## Figures and Tables

**Table 1:** Soil samples collected from apple-growing areas of Kayseri Province.

Province	EPN-Positive Samples	EPN-Negative Samples	Total	Recovery Rate (%)
Develi	4	26	30	13.3
Yahyalı	3	27	30	10.0
Yeşilhisar	5	35	40	14.2
Total	12	88	100	12

**Table 2:** Morphometric measurements of infective juveniles (IJs) of some of the isolates of entomopathogenic nematode species recovered from survey studies (μM).

Isolates	*L	*MBW	*EP	*ES	*TL	*a	*b	*c	*%D	*%E
*Steinernema feltiae*, Mean ± SE (Min–max) (n = 20)										
A67	861 ± 54 (805–896)	33 ± 2.6 (27–35)	59 ± 3.8 (54–65)	145 ± 4.1 (133–152)	81 ± 2.9 (75–83)	27 ± 3.3 (23–32)	7.5 ± 1.6 (5.9–7.1)	12.5 ± 2.2 (9.4–13.1)	41 ± 2.3 (37–44)	74 ± 3.2 (69–77)
A93	925 ± 61 (855–971)	31 ± 1.9 (27–33)	60 ± 4.1 (58–67)	126 ± 7.3 (119–142)	85 ± 4.5 (77–89)	29 ± 3.1 (26–33)	6.9 ± 0.9 (5.8–7.4)	11.1 ± 1.5 (9.5–11.4)	42.3 ± 2.9 (39–45)	74 ± 3.9 (70–78)
Nguyen and Hunt (2007)	879 ± 49 (766–928)	29 ± 1.9 (26–32)	63 ± 2.3 (59–67)	136 ± 3.5 (130–143)	86 ± 2.6 (81–89)	30 ± 1.9 (27–34)	6.4 ± 0.3 (5.8–6.8)	10 ± 0.5 (9.4–11)	-	-
*Heterorhabditis bacteriophora*, Mean ± SE (Min–max) (n = 20)										
A8	611 ± 8.1 (601–620)	21 ± 1.9 (18–24)	115 ± 8.2 (101–127)	145 ± 9.1 (132–155)	87.5 ± 3.7 (82–93)	24.1 ± 1.9 (21–27)	4.9 ± 0.9 (3.9–5.1)	6.9 ± 0.8 (6.6–7.8)	81 ± 3.1 (75–85)	104 ± 4.1 (99–112)
A20	597 ± 8.5 (588–609)	22 ± 2.5 (18–24)	105 ± 7.3 (96–117)	132 ± 4.9 (126–138)	90.7 ± 4.5 (83–96)	23.3 ± 2.7 (20–27)	4.5 ± 0.6 (4.1–4.8)	7.1 ± 0.9 (6.2–8.2)	82 ± 3.5 (77–86)	119 ± 6.5 (112–128)
Nguyen and Hunt (2007)	588 (512–671)	23 (18–31)	103 (87–110)	125 (100–139)	98 (83–112)	25 (17–30)	4.5 (4.0–5.1)	6.2 (5.5–7.0)	84 (76–92)	112 (103–130)

* Total body length (L), body length/body width (a), body length/esophageal length (b), body length/tail length (c), %E:[(EP/TL) × 100], %D:[(EP/ES) × 100], excretory pore (EP), pharynx (ES), maximum body width (MBW), tail length (TL), n: number of specimens examined.

**Table 3:** Entomopathogenic nematode isolates found, with the indication of the locality and internal transcribed spacer (ITS) sequence information.

Code	Location	District	Species	Accession Number
A6	38°19′50 K 35°22′52 D	Develi	*Steinernema feltiae*	OM401705
A8	38°21′02 K 35°23′11 D	Develi	*Heterorhabditis bacteriophora*	OM401700
A9	38°24′18 K 35°26′57 D	Develi	*Heterorhabditis bacteriophora*	OM401701
A20	38°24′18 K 35°26′45 D	Develi	*Heterorhabditis bacteriophora*	OM401702
A52	38°20′12 K 35°27′29 D	Yahyalı	*Heterorhabditis bacteriophora*	OM401704
A53	38°19′86 K 35°22′86 D	Yahyalı	*Steinernema feltiae*	OM401706
A54	38°42′58 K 35°31′89 D	Yahyalı	*Steinernema feltiae*	OM401707
A67	38°09′33 K 35°22′01 D	Yeşilhisar	*Steinernema feltiae*	OM401709
A93	38°24′18 K 35°26′57 D	Yeşilhisar	*Heterorhabditis bacteriophora*	OM401703
A94	38°21‘84 K 35°05‘64 D	Yeşilhisar	*Steinernema feltiae*	OM401708
A97	38°09′52 K 35°21′56 D	Yeşilhisar	*Steinernema feltiae*	OM401710
A100	38°20‘53 K 35°05‘59 D	Yeşilhisar	*Steinernema feltiae*	OM401711

**Table 4:** Mortality rates (%) of isolated entomopathogenic nematodes on the last instar larvae of *Galleria mellonella* at concentrations of 100 infective juveniles/Petri dish (100 IJs/Petri) under laboratory conditions (25 °C, 60% RH).

EPNs *	24 h	48 h	72 h
	(Mean ± S. Error)		
*S. feltiae* A6	23.3 ± 6.6 BC ^a^	43.3 ± 3.3 BC	60.0 ± 5.7 A
*H. bacteriophora* A8	16.6 ± 3.3 ABC	56.6 ± 6.6 CD	86.6 ± 8.8 ABC
*H. bacteriophora* A9	33.3 ± 3.3 C	66.6 ± 3.3 D	96.6 ± 3.3 BC
*H. bacteriophora* A20	13.3 ± 3.3 AB	43.3 ± 3.3 BC	86.6 ± 3.3 ABC
*S. feltiae* A52	16.6 ± 3.3 ABC	43.3 ± 3.3 BC	83.3 ± 3.3 ABC
*S. feltiae* A53	6.6 ± 3.3 AB	26.6 ± 3.3 AB	63.3 ± 6.6 A
*S. feltiae* A54	70.0 ± 5.7 D	96.6 ± 3.3 E	100.0 ± 0.0 C
*S. feltiae* A67	0.0 ± 0.0 A	23.3 ± 3.3 A	60.0 ± 10.0 A
*H. bacteriophora* A93	0.0 ± 0.0A	26.6 ± 3.3 AB	70.0 ± 5.7 AB
*S. feltiae* A94	60.0 ± 0.0 D	73.3 ± 3.3 D	86.6 ± 3.3 ABC
*S. feltiae* A97	13.3 ± 3.3 AB	43.3 ± 3.3 BC	86.6 ± 3.3 ABC
*S. feltiae* A100	16.6 ± 3.3 ABC	43.3 ± 3.3 BC	83.3 ± 3.3 ABC

* EPNs: Entomopathogenic nematodes. ^a^ Different capital letters show statistically significant differences among entomopathogenic nematode species/strains for each exposure time level.

**Table 5:** Repeated measures analysis of variance parameters for the main effects and associated interactions for mortality rates of the 5th instar larvae of *Cyida pomonella* in Petri dish bioassay.

Sources *	df	F-Value	*p*-Value
Nematodes (N)	3	4.605	0.007
Concentrations (C)	3	15.478	0.000
N*C	9	0.767	0.647
Error ^1^	48		
Exposure time (t)	3	371.153	0.000
C*t	9	5.984	0.000
N*t	9	3.987	0.000
N*C*t	27	1.071	0.382
Error ^2^	144		

Error ^1^ was used for comparing the mean levels of non-repeating factors when the interaction of the non-repeating factor with the repeated factor was not important. Error ^2^ was used for comparing the mean levels of the repeated factor. * Tukey (*p* ≤ 0.05).

**Table 6:** The efficacy of the selected EPN species/isolates on the 5th instar larvae of *C. pomonella* (25 °C, 60% RH).

Concentrations	Exposure Time * (h)	Mortality Rates (%)			
		*S. f.* A94	*S. f.* A54	*H. b.* A8	*H. b.* A9
10 IJs/larva	24	15.0 ± 9.5 A ^a^ a ^b^	10.0 ± 5.7 Aa	15.0 ± 5.0 Aa	10.0 ± 5.7 Aa
	48	35.0 ± 15.0 Ba	35.0 ± 5.0 Ba	30.0 ± 5.7 Ba	30.0 ± 5.7 Ba
	72	45.0 ± 5.0 Ba	40.0 ± 5.7 Ba	50.0 ± 5.7 Ca	40.0 ± 8.1 Ba
	96	65.0 ± 12.5 Ca	50 ± 5.7 Ba	60.0 ± 8.1 Ca	50.0 ± 5.7 Ba
25 IJs/larva	24	50.0 ± 12.9 Ab	35.0 ± 5.0 Aa	55.0 ± 9.5 Ab	50.0 ± 5.7 Ab
	48	60.0 ± 14.1 Aa	55.0 ± 9.5 Ba	75.0 ± 9.5 Bb	65.0 ± 9.5 Ba
	72	85.0 ± 9.5 Bb	60.0 ± 5.7 Ba	80.0 ± 8.1 Bb	75.0 ± 9.5 Bb
	96	90.0 ± 10.0 Bb	70.0 ± 5.7 Ba	85.0 ± 9.5 Bb	85.0 ± 9.5 Bb
50 IJs/larva	24	70.0 ± 10.0 Aa	70.0 ± 5.7 Aa	90.0 ± 10.0 Ab	90.0 ± 10.0 Ab
	48	80.0 ± 8.1 Aa	80.0 ± 8.1 Aa	95.0 ± 5.0 Ab	100.0 ± 0.0 Ab
	72	90.0 ± 5.7 ABa	85.0 ± 9.5 Aa	100.0 ± 0.0 Aa	100.0 ± 0.0 Aa
	96	95.0 ± 5.0 Ba	90.0 ± 5.7 Aa	100.0 ± 0.0 Aa	100.0 ± 0.0 Aa
100 IJs/larva	24	80.0 ± 8.1 Aa	95.0 ± 5.0 Ab	100.0 ± 0.0 Ab	95.0 ± 5.0 Ab
	48	90.0 ± 5.7 Aba	100.0 ± 0.0 Aa	100.0 ± 0.0 Aa	100.0 ± 0.0 Aa
	72	95.0 ± 5.0 Aba	100.0 ± 0.0 Aa	100.0 ± 0.0 Aa	100.0 ± 0.0 Aa
	96	100.0 ± 0.0 Ba	100.0 ± 0.0 Aa	100.0 ± 0.0 Aa	100.0 ± 0.0 Aa

* *S. f.* A94: *Steinernema feltiae* A94 isolate; *S. f.* A54: *Steinernema feltiae* A54 isolate; *H. b.* A8: *Heterorhabditis bacteriophora* A8 isolate; *H. b.* A9: *H. bacteriophora* A9 isolate. ^a^ Different capital letters show statistically significant differences among entomopathogenic nematode species/strains for each exposure time level. ^b^ Different lowercase letters show statistically significant differences among the exposure time levels for each entomopathogenic nematode species (*p* < 0.05 Tukey).

**Table 7:** Repeated measures analysis of variance parameters for the main effects and associated interactions for mortality rates of the larvae of *Cydia pomonella* in contact efficacy bioassay of cell-fee supernatants of symbiotic bacteria.

Sources *	df	F-Value	*p*-Value
Supernatant (S)	3	30.727	0.000
Error ^1^	12		
Exposure Time (t)	2	144.907	0.000
S*t	6	6.860	0.000
Error ^2^	24		

Error ^1^ was used for comparing the mean levels of non-repeating factors when the interaction of the non-repeating factor with the repeated factor was not important. Error ^2^ was used for com-paring the mean levels of the repeated factor. * Tukey (*p* ≤ 0.05).

**Table 8:** The mortality rates (%) of the 5th instar larvae of *Cydia pomonella* in contact efficacy bioassay of cell-fee supernatants of symbiotic bacteria of the selected entomopathogenic species/isolates.

Symbiotic Bacteria	Hours (h) after Treatment		
	24 h	48 h	72 h
X. b. A54	25.0 ± 2.5 A ^a^ a ^b^	40.0 ± 2.5 Ab	55.0 ± 5.0 Bc
X. b. A94	12.5 ± 3.1 Ba	50.0 ± 4.1 Ab	62.5 ± 4.1 Bb
P. l. A8	10.0 ± 3.8 Ba	30.0 ± 2.5 Ab	40.0 ± 2.5 Ab
P. l. A9	5.0 ± 2.5 Ba	15.0 ± 3.8 Bab	25.0 ± 3.8 Ab

X. b. A54 and X. b. A94: *Xenorhabdus bovienii*, P. l. A8 and P. l. A9: *Photorhabdus luminescens* subsp. *kayaii*. ^a^ Different capital letters show statistically significant differences among symbiotic bacteria species/strains for each exposure time level. ^b^ Different lowercase letters show statistically significant differences among the exposure time levels for each entomopathogenic nematode species (*p* < 0.05, Tukey).

## Data Availability

Data generated in this study are available upon reasonable request to the corresponding author.
